# Unveiling the Silent Invader: A Case Report on Miliary Tuberculosis

**DOI:** 10.7759/cureus.41817

**Published:** 2023-07-13

**Authors:** Spandana M, Seema Yelne, Minakshi Chaudhary, Aman Agrawal

**Affiliations:** 1 Paediatrics, Jawaharlal Nehru Medical College, Datta Meghe Institute of Higher Education and Research, Wardha, IND; 2 Nursing, Shalinitai Meghe College of Nursing, Datta Meghe Institute of Higher Education and Research, Wardha, IND; 3 Nursing, Shalinitai Meghe College of Nursing, Datta Meghe Institute of Higher Education and Resesrach, Wardha, IND; 4 Medicine, Jawaharlal Nehru Medical College, Datta Meghe Institute of Higher Education and Research, Wardha, IND

**Keywords:** afb, igra, immunosuppression, x-ray, miliary tuberculosis

## Abstract

Miliary tuberculosis (TB) is characterized by the spreading of *Mycobacterium tuberculosis* throughout the body, leading to various clinical manifestations and potential complications. This case involves a 58-year-old male who presented with fever, night sweats, weight loss, and respiratory symptoms. Diagnostic workup revealed the characteristic radiological findings of diffuse miliary nodules on CT scan and X-ray of the chest. Laboratory investigations, including a positive interferon-gamma release assay, supported the diagnosis. The patient was initiated on a multidrug anti-TB regimen consisting of rifampicin, isoniazid, pyrazinamide, and ethambutol, with adjunctive corticosteroids for severe manifestations. Close monitoring and supportive care were provided. The patient started anti-TB therapy and his health improved significantly. He was able to receive a kidney transplant successfully. The case report emphasizes the importance of early recognition, timely diagnosis, and appropriate treatment initiation to improve patient outcomes.

## Introduction

Miliary tuberculosis (TB) is a rare and severe form of TB characterized by the widespread dissemination of *Mycobacterium tuberculosis* throughout the body [1]. It is named after the millet seed-sized lesions that resemble tiny millet seeds scattered throughout affected organs, particularly the lungs. Miliary TB occurs when the mycobacteria access the bloodstream, allowing them to spread to various organs and tissues [2,3].

Miliary TB poses significant diagnostic and therapeutic challenges due to its diverse clinical manifestations and the potential for rapid disease progression. While the incidence of miliary TB has decreased in many developed countries, it remains a significant concern in regions with high TB prevalence and among immunocompromised populations [4].

Miliary TB presents a broad spectrum of clinical manifestations, such as fever, night sweats, weight loss, fatigue, and a persistent dry cough. These symptoms are similar to those of other systemic diseases, leading to delayed or missed diagnoses. Additional symptoms may include respiratory distress, hepatosplenomegaly, neurological abnormalities, and skin findings. It is important to consider miliary TB in the differential diagnosis of patients with compatible clinical features, especially in those with risk factors such as recent travel to endemic regions or immunosuppression [5,6].

## Case presentation

We present a case of a 58-year-old male who was admitted to our hospital with a 2-month history of fever, night sweats, weight loss of 10 kg, and progressive dyspnea. He had no history of TB exposure or contact, no previous TB treatment, and no known immunodeficiency or comorbidity. On physical examination, he was febrile (38.5°C), tachycardic (110 beats/min), tachypneic (28 breaths/min), and hypoxic (SpO_2_ 90% on room air). He had bilateral crackles on chest auscultation and hepatosplenomegaly on abdominal palpation. No lymphadenopathy, skin lesions, or neurological deficits were noted. The initial laboratory tests showed (Table 1).

**Table 1:** Laboratory investigation ESR: erythrocyte sedimentation rate CRP: C-reactive protein

Investigation	Patient value	Reference value
Hemoglobin	9.8 g/dL	13.5 g/dL to 15.5 g/dL
White blood cell count	12.4 x 10^9/L	4.5 to 11.0 × 109/L
Platelet count	450 x 10^9/L	150 x 10^9/L to 400 x 10^9/L
ESR	12 mm/h	0 to 30 mm/hr
CRP	1 mg/L	0.3 to 1 mg/dL
Serum albumin	2.8 g/dL	3.5 to 5.3 mg/dL

**Figure 1:**
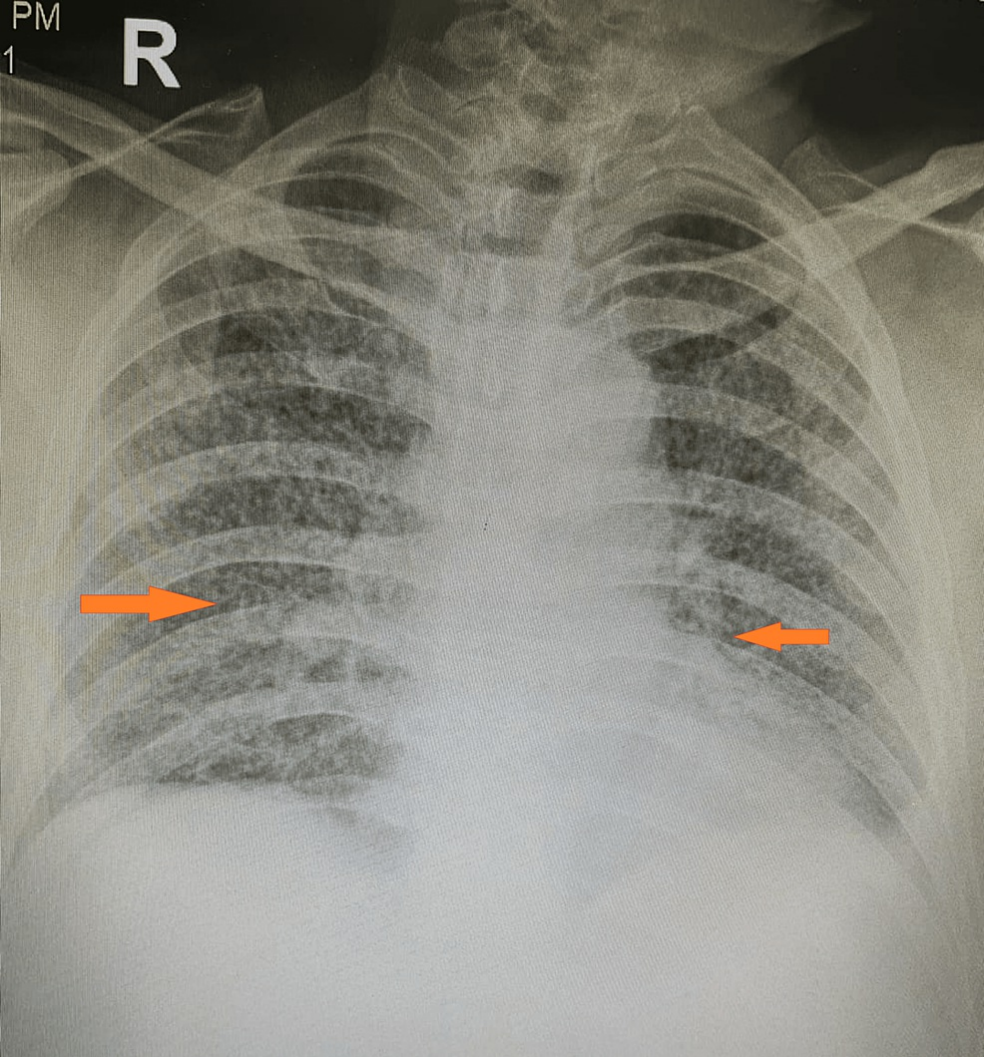
Chest X-ray showing diffuse miliary nodules throughout both lung fields

**Figure 2:**
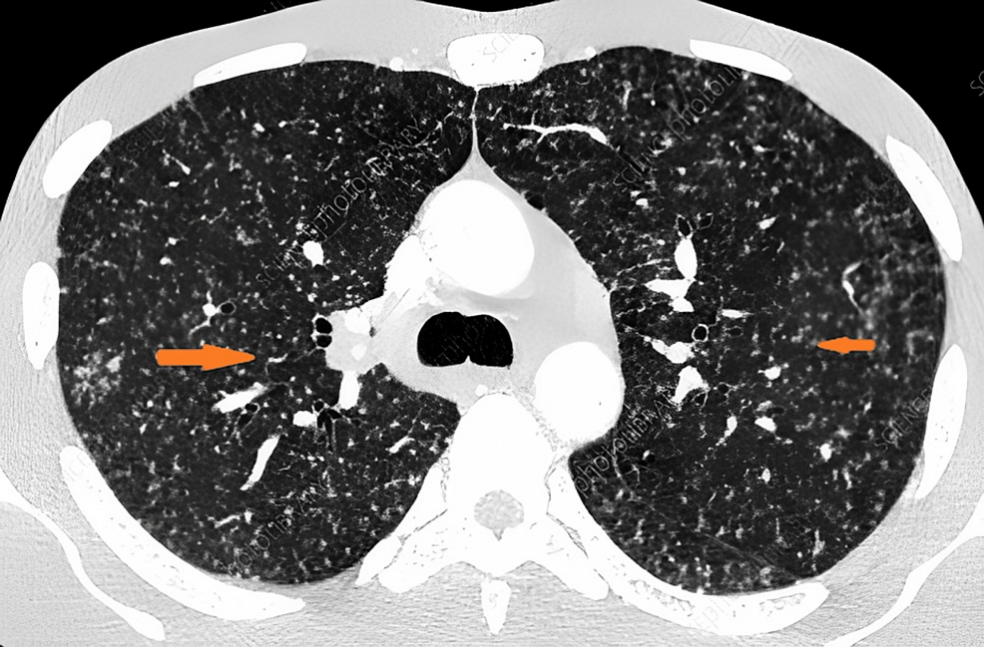
Chest CT scan revealed numerous tiny nodules scattered throughout both lungs

The liver and renal function tests were normal. The tuberculin skin test (TST) was negative, but the interferon-gamma release assay (IGRA) was positive. The HIV test was negative. The sputum smear for acid-fast bacilli (AFB) was negative. The chest X-ray and CT scan showed diffuse miliary nodules in both lungs (Figure 1). The abdominal ultrasound revealed multiple hypoechoic nodules in the liver and spleen (Figure 2). A lumbar puncture was done and cerebrospinal fluid (CSF) was sent for staining and culture for AFB. The CSF analysis showed a lymphocytic pleocytosis (150 cells/mm^3), elevated protein (80 mg/dL), and low glucose (40 mg/dL). The CSF culture for AFB was negative. The bone marrow biopsy showed granulomatous inflammation with caseation necrosis, but no AFB was seen on the Ziehl-Neelsen stain. The CSF was positive for AFB.

Based on the clinical and radiological findings, the diagnosis of miliary TB was made and the patient was started on a four-drug anti-TB regimen consisting of rifampicin 600 mg, isoniazid 300 mg, pyrazinamide 1.5 g, and ethambutol 800 mg daily, along with pyridoxine 25 mg daily. He was also given prednisolone 40 mg daily for the first 4 weeks, then tapered by 10 mg every week, for the management of respiratory distress and possible TB meningitis. He was admitted to the isolation ward and received oxygen therapy, intravenous fluids, and nutritional support. He showed gradual improvement in his symptoms and oxygen saturation over the next few weeks. The blood culture for *M. tuberculosis* became positive after 4 weeks of treatment, confirming the diagnosis. The urine and sputum cultures remained negative. The drug susceptibility testing showed no resistance to any of the first-line anti-TB drugs. The patient completed the intensive phase of treatment for 2 months and was switched to the continuation phase of rifampicin and isoniazid for another 4 months. He was discharged from the hospital after 2 months of treatment and followed up at the outpatient clinic regularly. He had no adverse effects from the anti-TB drugs or corticosteroids. He regained his weight and appetite and had no fever or respiratory symptoms.

The management plan included initiating anti-TB therapy and supportive care, nutritional support, and glucose control for diabetes management. The patient was admitted to the hospital for close monitoring and initiation of treatment. Regular follow-up visits were scheduled to assess treatment response, monitor the adverse effects of medications, and ensure treatment adherence.

## Discussion

Miliary TB is a rare and severe form of tuberculosis characterized by disseminating *M. tuberculosis* throughout the body. This case report highlights the challenges associated with diagnosing and managing miliary TB and the importance of early recognition and appropriate treatment initiation.

The patient presented with classic symptoms of miliary TB, including fever, night sweats, weight loss, and respiratory symptoms [6]. These nonspecific symptoms can overlap with acute respiratory distress syndrome, Addison's disease, ascites, and other systemic diseases, challenging diagnosis. In this case, risk factors such as recent travel to an endemic region and compatible clinical features prompted further investigation for TB.

Radiological imaging, including chest X-rays and CT scans, is crucial in confirming the diagnosis. The characteristic findings of diffuse miliary nodules throughout the lungs were consistent with miliary TB. These radiological features are important to recognize, as they can help differentiate miliary TB from other pulmonary diseases with similar clinical presentations [7,8].

Laboratory investigations for miliary TB can be challenging due to the paucibacillary nature of the disease. Acid-fast bacilli (AFB) smears and cultures are often negative. However, the positive IGRA supported the diagnosis by detecting the patient's immune response to *M. tuberculosis* infection [9]. This highlights the importance of utilizing alternative diagnostic methods when traditional tests yield negative results.

The management of miliary TB involves the prompt initiation of appropriate anti-TB therapy. In this case, the patient was started on a multidrug regimen consisting of rifampicin, isoniazid, pyrazinamide, and ethambutol. Additionally, adjunctive corticosteroids were administered due to the severity of the patient's symptoms. Close monitoring and supportive care were provided to manage potential complications and improve treatment outcomes [10].

Miliary TB carries a high risk of complications and mortality if left untreated. The dissemination of *M. tuberculosis* throughout the body can lead to severe organ dysfunction, respiratory failure, and disseminated intravascular coagulation. Therefore, early diagnosis and treatment initiation are critical in improving patient outcomes [10].

The challenges in diagnosing and managing miliary TB underscore the need for increased awareness among healthcare professionals. Maintaining a high index of suspicion is crucial, especially in patients with risk factors or compatible clinical features. Moreover, continued research is necessary to develop improved diagnostic methods that can provide more rapid and accurate results, enabling earlier intervention and treatment initiation.

## Conclusions

In this 58-year-old case, chest X-ray and chest CT scan findings were consistent with miliary TB. However, there was no evidence of cavitation or lymphadenopathy, the diagnosis was confirmed on the basis of history, clinical presentation, laboratory findings, positive IGRA, and characteristic radiological findings. A multidisciplinary approach involved infectious disease specialists, pulmonologists, and other relevant healthcare providers for optimal management and improved patient outcomes.
